# Real-world clinical outcomes of EKOS catheter-directed thrombolysis versus systemic alteplase in acute pulmonary embolism: a retrospective cohort study

**DOI:** 10.3389/fphar.2025.1709708

**Published:** 2026-01-06

**Authors:** Tarek Mahmoud Senosy, Asmaa A. Elsayed, Eman Fathi Abd Razik, Tarek Khairy Mosa, Ahmed Mahmoud Ali, Ahmed R. N. Ibrahim, Osama Nady Mohamed, Engy A. Wahsh

**Affiliations:** 1 Cardiology Department, Faculty of Medicine, Minia University, Minia, Egypt; 2 Clinical Pharmacy Department, Faculty of Pharmacy, Sohag University, Sohag, Egypt; 3 Department of Public Health, Faculty of Medicine, Minia University, Minia, Egypt; 4 Department of Cardiology, Faculty of Medicine, Ain Shams University, Cairo, Egypt; 5 Department of Clinical Pharmacy, College of Pharmacy, King Khalid University, Abha, Saudi Arabia; 6 Department of Internal Medicine, Faculty of Medicine, Minia University, Minia, Egypt; 7 Clinical Pharmacy Department, Faculty of pharmacy, October 6 University, Giza, Egypt

**Keywords:** EKOS, alteplase, pulmonary embolism, thrombolysis, anticoagulants

## Abstract

**Background:**

The use of EkoSonic Endovascular System (EKOS) that combines ultrasound technology with catheter-directed thrombolysis has shown promising outcomes in patients with pulmonary embolism (PE) in dissolving clots effectively. This method targets thrombus removal precisely, minimizing systemic exposure to thrombolytic agents and thereby reducing bleeding risks. Given its minimally invasive approach and focused delivery, EKOS offers a safer alternative for intermediate high-risk patients in whom traditional anticoagulation or systemic thrombolysis may pose higher risks of complications. This study compares the safety and effectiveness of EKOS versus intravenous (IV) alteplase infusion in managing acute PE.

**Methods:**

A retrospective cohort study was conducted at a tertiary care hospital, examining patient records from 2022 to 2024 according to predefined eligibility criteria. Individuals aged 18 years or older diagnosed with intermediate- to high-risk PE, who showed clinical deterioration after 48 h of anticoagulation, were enrolled, as assessed by the National Early Waning Score (NEWS). Patients were excluded if they had a stable PE or were at a high risk of hemodynamic decompensation at the time of presentation. The primary outcomes were the change in tricuspid annular plane systolic excursion (TAPSE) and right ventricular-to-left ventricular (RV/LV) diameter ratio from baseline to 1-week outpatient follow-up.

**Results:**

Out of 104 eligible patients, 54 received EKOS (EKOS group) and 50 received IV alteplase infusion (control group). The RV/LV diameter ratio significantly decreased, while TAPSE improved significantly in the EKOS and control groups. At the end of follow-up, the incidence of major bleeding events within 72 h was significantly lower in the EKOS group (2 vs. 8; p = 0.03). Although all-cause mortality at 6 months was lower in the EKOS group, the difference was not statistically significant (p = 0.3).

**Conclusion:**

EKOS showed comparable efficacy to systemic thrombolysis, reducing right heart strain, improving RV function, and minimizing complications in patients with intermediate- to high-risk PE after failed anticoagulation, with deterioration in clinical condition and the need for rescue thrombolysis. In addition, EKOS had a lower incidence of major bleeding within 72 h, making it a safer option for rescue thrombolysis.

## 1 Introduction

Pulmonary embolism (PE) is a serious cardiovascular condition where a blood clot, typically originating in the deep veins of the legs, obstructs a pulmonary artery in the lungs, thus impairing blood flow and potentially causing severe respiratory distress, chest pain, and even sudden death. It is commonly associated with deep vein thrombosis (DVT) and is often part of a larger condition known as venous thromboembolism (VTE) (Freund et al., 2022).

PE presents significant morbidity and mortality risks as it can lead to severe respiratory compromise, cardiac strain, and sudden death if left untreated. Mortality rates for untreated PE are high, with acute cases contributing to a substantial proportion of cardiovascular deaths. Even with treatment, morbidity remains a concern due to potential long-term complications, such as chronic thromboembolic pulmonary hypertension (CTEPH). Early detection and timely intervention are essential in reducing both mortality and the burden of chronic complications associated with PE (Gottlieb et al., 2024).

Systemic thrombolysis is a treatment for PE primarily reserved for high-risk cases with hemodynamic instability, where the benefits of rapid clot dissolution outweigh the risks of bleeding complications. Administered intravenously, thrombolysis can effectively restore blood flow in severe PE cases but carries a high risk of major hemorrhage, particularly intracranial bleeding (Zhang et al., 2023).

For patients who are not candidates for thrombolysis due to bleeding risk, alternative interventions are available. Pulmonary embolectomy, a surgical option, directly removes the clot but is typically used in life-threatening cases or when other methods are contraindicated (Götzinger et al., 2023).

Extracorporeal membrane oxygenation (ECMO) offers respiratory and circulatory support in critical PE cases, especially when combined with other clot-removal techniques (Boey et al., 2023).

Additionally, catheter-directed thrombolysis (CDT) has emerged as a less invasive, and targeted approach, allowing for localized thrombolytic delivery or mechanical clot disruption, with a generally lower bleeding risk compared to systemic thrombolysis. The choice of intervention is based on specific risk factors in addition to patient stability (Salinas et al., 2024).

FlowTriever and Indigo, catheter-based embolectomy systems, have been designed for mechanical clot removal without the use of thrombolytics. The FlowTriever system, for instance, uses a catheter to aspirate clots from the pulmonary arteries, providing immediate physical removal of embolus. It has been particularly effective for patients at high risk of bleeding who cannot receive thrombolytic drugs. Similarly, the Indigo system by Penumbra uses continuous aspiration technology to capture and remove embolic material and thus allows direct mechanical clot removal (Elmoghrabi et al., 2023; Raza et al., 2023).

The EkoSonic Endovascular System, developed by EKOS Corporation, is a specialized CDT system that combines ultrasound technology with thrombolytic infusion to treat PE. This dual mechanism is designed to enhance the penetration of the thrombolytic agent into the clot, thereby enhancing clot breakdown while using lower doses of thrombolytic drugs. The system’s catheter consists of multiple side ports for delivering a continuous, controlled dose of thrombolytic medication, while ultrasound waves facilitate the creation of microchannels within the thrombus (Lumsden and Suarez, 2016; Finocchiaro et al., 2024).

Studies have found that ultrasound-assisted thrombolysis, provided by the EkoSonic system, reduces the amount of tissue plasminogen activator (tPA) required, significantly lowering the risk of bleeding compared with systemic thrombolysis. As a result, the EkoSonic system is often chosen for patients with intermediate- to high-risk PE who need thrombolytic therapy but present a heightened risk of major bleeding (Kennedy et al., 2023).

Additionally, the system enables continuous monitoring, allowing clinicians to adjust the thrombolytic dosage to optimize patient outcomes (Choksi et al., 2025). Although these trials demonstrated promising results, their evaluation of the EKOS intervention’s safety and effectiveness was limited to the immediate post-treatment period. Data regarding the long-term impact of EKOS therapy are insufficient. Therefore, our study aims to investigate the safety and effectiveness of EKOS compared to that of IV alteplase infusion in patients with PE.

## 2 Patients and methods

A retrospective cohort study conducted at a tertiary care hospital reviewed patients’ records according to previously established eligibility criteria.

The current study was conducted in accordance with the guidelines of the Declaration of Helsinki and was approved by the Institutional Review Board of Saudi German Hospital (No. SGHJ-IRB-045). Due to its retrospective design, individual informed consent was deemed not applicable and was waived by the ethics committee.

### 2.1 Inclusion criteria

- Patients ≥ 18 years diagnosed with massive or sub-massive acute PE.
- Intermediate- to high-risk probability for having adverse outcomes in PE was defined as having RV dysfunction (detected via computed tomography (CT) scan or echocardiography), with signs such as RV enlargement, RV hypokinesis, or higher RV/LV diameter ratio (>0.9–1.0).
- Elevated cardiac biomarkers such as troponin or brain natriuretic peptide (BNP), which indicate strain or injury to the heart muscle.
- Deterioration in clinical condition after 48 h of anticoagulation according to the NEWS score was determined for all patients according to the following parameters: respiratory rate, SPO2%, any supplemental oxygen needed, temperature, systolic blood pressure (mmHg), pulse rate, and the level of consciousness.

### 2.2 Exclusion criteria

Patients who presented with stable PE and a high risk of hemodynamic decompensation at the presentation time.

### 2.3 Data collection and grouping

Patient electronic medical records from 2022 to 2024 were investigated according to the prior criteria, and then, patients were categorized into the following:

EKOS group: patients received alteplase via EKOS with doses described in Table 2.

Control group: patients with PE who received a 100-mg IV alteplase infusion over 2 h.

### 2.4 Sample size calculations

The sample size was determined based on the primary echocardiographic outcome, that is, the change in TAPSE from baseline to 1 week after treatment. Previous studies evaluating the efficacy of ultrasound-assisted catheter-directed thrombolysis (USAT/EKOS) in patients with intermediate- to high-risk PE have reported an improvement in mean TAPSE of approximately 3 mm–4 mm, with a standard deviation of approximately 3.5 mm (Kucher et al., 2014; Al-Terki et al., 2023; Colombo et al., 2024). Assuming a clinically meaningful difference of 2 mm between the treatment groups, with a two-sided significance level of 0.05% and 80% statistical power, the required sample size was estimated at 48 patients per group. To account for potential loss to follow-up or incomplete echocardiographic data, the number of patients enrolled was increased to 55 per group.

This retrospective cohort included a total of 104 patients (54 in the EKOS group and 50 in the intravenous alteplase group), providing an achieved power of approximately 83% to detect the prespecified difference in TAPSE.

### 2.5 Study outcomes

The following parameters were collected: patient’s demographics, past clinical history, family history of VTE, comorbidities, and immobility or surgery within 30 days of PE presentation.

Information was also recorded regarding the medication history, especially the thrombolytics administered via EKOS, the pre-procedure anticoagulant, the anticoagulant offered at discharge, and inotropes or vasopressors used to stabilize blood pressure.

#### 2.5.1 Primary outcome measure

The difference in the RV/LV diameter ratio from baseline to the initial outpatient follow-up was recorded using an echocardiogram (ECG). Doppler ECG estimated the RV velocity as the peak speed of the tricuspid valve’s movement toward the heart apex during contraction (lower values than 0.1 m/s are correlated to impaired RV systolic function). In addition, the TAPSE was measured.

#### 2.5.2 Secondary outcome measures

1. All-cause 30-day readmission rate and all-cause mortality at 6 months.
2. Major bleeding within 72 h and 6 months.

Major bleeding was defined as symptomatic bleeding resulting in a decrease in the hemoglobin level of ≥ 2 g/dL or necessitating the transfusion of ≥ 2 units of red blood cells or whole blood.

### 2.6 Statistical analysis

Data entry and statistical analysis were performed with SPSS v 20.0 software (IBM Corp., Armonk, NY, USA). Quantitative variables were presented as mean ± standard deviation (SD). Categorical variables were presented as numbers and percentages (%). The chi-square and independent t-test were used to measure statistical significance between the participant groups. A two-tailed p-value less than 0.05 was considered significant.

Risk ratios (RRs) with corresponding 95% confidence intervals (CIs) were calculated to compare event rates between groups. The 95% CIs for proportions were derived using the exact Clopper–Pearson method. Univariate logistic regression analyses were performed to explore the potential predictors of major bleeding and all-cause mortality at 6 months, including demographic, hemodynamic, and echocardiographic variables (age, sex, BMI, systolic and diastolic blood pressure, comorbidities, timing to therapy, RV/LV ratio, TAPSE, RV velocity, and treatment modality). Odds ratios (ORs) with 95% CIs and *P*-values were reported for each variable.

## 3 Results

**FIGURE 1:**
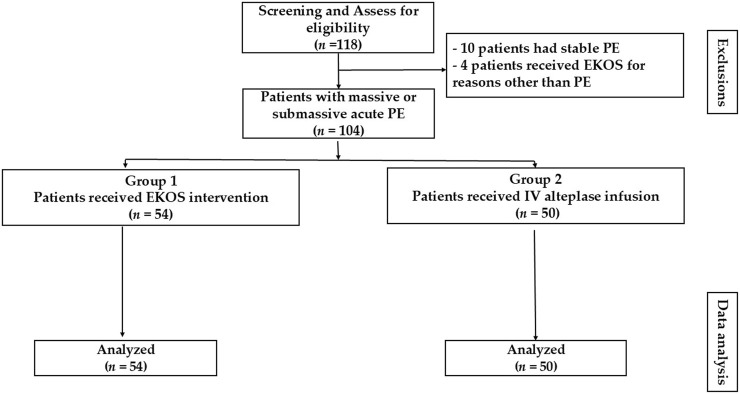
Group selection flowchart.

During the study period, 104 patients were identified from the records as having acute PE and met the eligibility criteria. From the eligible records, 54 received EKOS (EKOS group) and 50 received IV alteplase infusion (control group). The reasons for exclusion included the following: undergoing EKOS procedures for indications unrelated to PE (n = 4) and having stable PE (n = 10) (Figure 1).

Parameters of NEWS SCORE after a 48-h follow-up of oral anticoagulant therapy in the EKOS and control groups were estimated according to the previously mentioned parameters. Comparisons between groups for the previously mentioned parameters yielded statistically insignificant results (see Supplementary Table 1).

### 3.1 Baseline characteristics

The average age of the EKOS group patients was 59.1 ± 5.8 years, and that of the control group participants was 57.5 ± 6.6 . A total of 24 (44.4%) patients were males and 30 (55.6%) patients were females in the EKOS group; 24 (44.4%) patients were males and 26 (55.6%) patients were females in the control group. Table 1 shows no significant difference in baseline parameters, except that patients who received EKOS had a higher mean TAPSE compared to the control group (P = 0.04).

**TABLE 1:** Baseline characteristics of the studied patients.

Parameters	EKOS cases (n = 54)	Alteplase controls (n = 50)	P-value
Age (years)	59.1 ± 5.8	57.5 ± 6.6	0.2
Sex			
Male	24 (44.4%)	24 (48.0%)	0.84
Female	30 (55.6%)	26 (52.0%)	
BMI (kg/m^2^)	37.9 ± 6.5	38 ± 7.2	0.2
Obesity	47 (87.0%)	43 (84.0%)	0.78
Family history of VTE	6 (11.1%)	5 (10.0%)	1
SBP	123.1 ± 18.1	122.9 ± 19.5	0.97
DBP	78.2 ± 9.7	78.1 ± 9	0.98
RV/LV ratio	1.4 ± 0.19	1.4 ± 0.17	0.48
Patient’s clinical history			
Cancer	3 (5.6%)	2 (4.0%)	0.71
History of VTE	13 (24.1%)	11 (22.0%)	0.82
Immobility ≤30 days	12 (22.2%)	10 (20.0%)	0.78
Surgery ≤30 days	5 (9.3%)	2 (4.0%)	0.29
Heart failure	4 (7.4%)	1 (2.0%)	0.91
COPD	6 (11.1%)	5 (10.0%)	0.85
Asthma	16 (29.6%)	11 (22.0%)	0.37
Use of hormone therapy	8 (14.8%)	8 (16.0%)	0.80
TAPSE (cm)	1.5 ± 0.20	1.4 ± 0.19	0.04*
Anticoagulant at discharge			
Apixaban	13 (24.1%)	12 (24.0%)	0.30
Rivaroxaban	24 (44.4%)	14 (28.0%)	
Warfarin	7 (13.0%)	8 (16.0%)	
Edoxaban	4 (7.4%)	11 (22.0%)	
Dabigatran	3 (5.6%)	3 (6.0%)	
Enoxaparin bridging therapy for dabigatran	3 (5.6%)	2 (4.0%)	
Time from presentation to anticoagulation (hours)	3.7 ± 0.81	3.8 ± 0.86	0.98

* RV, right ventricular; LV, left ventricular TAPSE, tricuspid annular plane systolic excursion; COPD, chronic obstructive pulmonary disease; VTE, venous thromboembolism; BMI, body mass index.

The most common anticoagulant at CCU admission and discharge in both groups was rivaroxaban. However, the least used anticoagulant was dabigatran and enoxaparin bridging therapy for dabigatran in EKOS patients, and enoxaparin bridging therapy for dabigatran in the control patients (Table 1).

Table 2 summarizes the characteristics of EKOS patients considering the total alteplase dose received, time from presentation to EKOS, and total procedure time.

**TABLE 2:** EKOS parameters.

EKOS cases (n = 54)	
Total dose of alteplase received (mg)	23.03 ± 3.5
Alteplase dose received via EKOS	
1-mg bolus, 1 mg/h over 12 h	22 (40.7%)
5-mg bolus, 0.5 mg/h over 12 h	26 (48.1%)
2.5-mg bolus, 0.5 mg/h over 12 h	6 (11.1%)
Anticoagulant prior to EKOS	
Heparin	39 (72.2%)
Enoxaparin	15 (27.8%)
Time from presentation to EKOS (hours)	21.8 ± 2.8
EKOS procedure time (min)	46.3 ± 6.2

### 3.2 Comparison between alteplase through EKOS and IV infusion of alteplase

Regarding the study outcomes, the RV/LV diameter ratio was significantly decreased in both the EKOS and control groups (p < 0.0001 and p < 0.0001, respectively), and the RV velocity was significantly increased in both the EKOS and control groups (p < 0.0001 and p < 0.0001, respectively). In addition, there was a significant improvement in TAPSE in both groups (p < 0.0001 and p < 0.0001, respectively).

However, there were no statistical differences between the two groups in the aforementioned parameters (Table 3).

**TABLE 3:** Comparison between alteplase through EKOS and IV infusion on RV dimensions, velocity, and function.

Parameters	​	EKOS cases	IV alteplase infusion controls	P-value between groups
RV/LV diameter ratio	Pre	1.4 ± 0.19	1.4 ± 0.17	0.19
	Post	0.96 ± 0.22	0.96 ± 0.22	
	P-value	<0.0001	<0.0001	
RV S′ velocity (cm/s)	Pre	0.12 ± 0.04	0.12 ± 0.05	0.12
	Post	0.15 ± 0.05	0.18 ± 0.07	
	P-value	<0.0001	<0.0001	
TAPSE (cm)	Pre	1.52 ± 0.19	1.44 ± 0.19	0.08
	Post	2.17 ± 0.38	2.00 ± 0.34	
	P-value	<0.0001	<0.0001	

RV, right ventricular; LV, left ventricular; TAPSE, tricuspid annular plane systolic excursion.

Regarding the end of follow-up outcomes of the studied groups, in the EKOS group, two (3.7%) patients needed further intervention after EKOS, two (3.7%) patients had major bleeding events within 72 h, two (3.7%) patients had major bleeding events within 6 months, and one patient (1.9%) died within 6 months after EKOS. In the control group, three (6.0%) patients needed further intervention, eight (16.0%) patients had major bleeding events within 72 h, two (4.0%) patients had major bleeding events within 6 months, and three patients (6.0%) died within 6 months. There was a significant difference between the two groups, as evidenced by major bleeding events within 72 h (p = 0.03) (Table 4).

**TABLE 4:** Comparison of end of follow up in secondary outcomes between EKOS and systemic alteplase

Outcome variable	Group	Events n/N (%)	95% CI for risk	Risk ratio (95% CI)	P-value
Major bleeding event within 72 h	EKOS	2/54 (3.7%)	0.9–13.8	0.23 (0.05–1.00)	0.03
	Alteplase	8/50 (16%)	8.3–28.7	-	
Major bleeding event within 6 months	EKOS	2/54 (3.7%)	0.9–13.8	0.93 (0.14–6.12)	0.94
	Alteplase	2/50 (4%)	1.1–13.5	-	
All-cause mortality at 6 months	EKOS	1/54 (1.9%)	0.3–9.9	0.31 (0.03–2.96)	0.35
	Alteplase	3/50 (6%)	2.1–16.3	​	
All-cause 30-day readmission	EKOS	2/54 (3.7%)	0.4–8.7	0.62 (0.11–3.36)	0.67
	Alteplase	3/50 (6%)	0.5–10.3	​	

### 3.3 Logistic regression analysis

The univariate logistic regression for mortality shows that most baseline demographic and hemodynamic variables, including age, sex, BMI, blood pressure, obesity, comorbidities, and baseline RV parameters, were not statistically significant predictors of death. The only significant factor was a decrease in hemoglobin levels within 72 h (OR = 10.33, 95% CI 1.44–74.13, p = 0.02), indicating that patients who experienced early bleeding or hemoglobin loss had a markedly higher risk of death. A trend toward higher mortality was observed among patients with major bleeding within 6 months (OR = 8.00, p = 0.10), but the CI was wide, suggesting limited power. No significant association of the treatment modality (EKOS vs. systemic alteplase) was not significantly associated with mortality (p = 0.59) was observed, confirming that both approaches produced comparable survival outcomes. Baseline echocardiographic markers (RV/LV ratio, TAPSE, and RV velocity) were not predictive of mortality in this cohort, possibly due to early correction of RV dysfunction after reperfusion therapy and the small number of deaths (four in total).

In the bleeding analysis, no demographic or clinical parameter showed a robust independent association with bleeding events. Most variables, including age, sex, BMI, blood pressure, comorbidities, and timing to therapy, were non-significant. Variables related to timing (time to anticoagulation or to EKOS) and drug-exposure fields (alteplase bolus and total dose) were also non-significant. A borderline association was observed for baseline TAPSE (p = 0.05, OR = 414.49, 95% CI 0.99–172131.05), but the extremely wide CI implies an unstable estimate that was likely due to few bleeding cases. The treatment group (EKOS vs. alteplase) was not statistically significant (p = 0.29), though numerically, the odds ratio > 1 suggests slightly more bleeding with systemic alteplase, which is consistent with our descriptive data (16% vs. 3.7%). Overall, the univariate model indicates that no baseline echocardiographic or clinical parameter were strong predictors of bleeding, except for possible spurious signals arising from data sparsity (Table 5).

**TABLE 5:** Univariate logistic regression analyses.

Variables	p-value	Odds ratio (OR)	95% CI for OR	
			Lower bound	Upper bound
A: To predict mortality in pulmonary embolism				
Age	0.90	0.99	0.86	1.14
Sex	0.26	0.28	0.03	2.56
BMI	0.87	0.99	0.87	1.13
Obesity	0.72	0.66	0.07	6.33
Family history of VTE	0.99	0.00	0.00	​
Systolic BP (mmHg)	0.95	1.002	0.95	1.05
Diastolic BP (mmHg)	0.74	0.98	0.89	1.08
Cancer	0.99	0.00	0.00	​
History of VTE	0.87	0.83	0.09	7.76
Recent immobility ≤30 days	0.95	0.93	0.09	8.75
Surgery ≤30 days	0.26	3.88	0.37	40.29
Heart failure	0.99	0.00	0.00	​
COPD	0.99	0.00	0.00	​
Hormonal use	0.99	0.00	0.00	​
Anticoagulant prior to EKOS	0.69	1.46	0.23	9.19
Time from presentation to EKOS	0.60	0.98	0.90	1.06
Time from presentation to anticoagulation	0.63	1.31	0.43	4.02
Baseline RV/LV ratio	0.51	5.51	0.04	879.39
Baseline RV velocity	0.62	160.16	0.00	8.70
Baseline TAPSE	0.55	0.24	0.002	25.38
Hb decrease within 72 h	0.02	10.33	1.44	74.13
Major bleeding within 6 months	0.10	8.00	0.67	94.99
EKOS vs. Alteplase	0.59	1.66	0.27	10.37
B: To predict bleeding risk in the studied groups				
Age	0.40	0.94	0.80	1.09
Sex	0.27	3.667	0.37	36.47
BMI	0.26	3.667	0.37	36.47
Obesity	0.55	0.488	0.05	5.03
Family history of VTE	0.99	0.00	0.00	​
Systolic BP (mmHg)	0.21	0.961	0.90	1.02
Diastolic BP (mmHg)	0.54	1.036	0.93	1.16
Cancer	0.99	0.00	0.00	​
History of VTE	0.22	3.545	0.47	26.63
Recent immobility ≤30 days	0.85	1.254	0.12	12.68
Surgery ≤30 days	0.99	0.00	0.00	​
Heart failure	0.99	0.00	0.00	​
COPD	0.99	0.00	0.00	​
Hormonal use	0.11	5.250	0.69	40.04
Alteplase bolus dose	0.24	0.35	0.06	1.99
Total dose of alteplase	0.31	1.02	0.99	1.05
Anticoagulant prior to EKOS	0.77	0.708	0.07	7.08
Time from presentation to EKOS	0.27	0.941	0.84	1.05
Time from presentation to anticoagulation	0.25	2.244	0.57	8.86
Baseline RV/LV ratio	0.94	0.81	0.004	186.89
Baseline RV velocity	0.47	3065.07	0.00	7178262427008.14
Baseline TAPSE	0.05	414.49	0.99	172131.05
Group (EKOS vs. Alteplase)	0.29	3.38	0.34	33.63

BMI, body mass index; CI, confidence interval; COPD, chronic obstructive pulmonary disease; EKOS, EkoSonic Endovascular System; Hb, hemoglobin; HF, heart failure; OR, odds ratio; RV/LV, right ventricular to left ventricular diameter ratio; RV velocity, right ventricular systolic velocity; TAPSE, tricuspid annular plane systolic excursion; VTE, venous thromboembolism.

## 4 Discussion

PE is the third leading cause of cardiovascular death worldwide, after myocardial infarction and cerebrovascular disorders (Khan et al., 2019).

Combining ultrasound technology with CDT in patients with PE has shown promising outcomes by dissolving clots more effectively. This technique targets the precise removal of thrombi, thereby reducing systemic exposure to thrombolytic agents and lowering the risk of bleeding (Draxler et al., 2025). Moreover, EKOS’s targeted delivery and minimally invasive nature provide a safer treatment option for high-risk patients, especially when conventional anticoagulation or systemic thrombolysis treatments carry a higher risk of complications (Khan et al., 2019).

The present study showed that patients in the EKOS and control groups had a significantly decreased RV/LV diameter ratio. At the same time, TAPSE was significantly improved, with slightly higher TAPSE in EKOS cases (2.17 vs. 2.00). Additionally, both groups experienced significant increases in RV systolic velocity, indicating improvements in RV function. These findings support the efficacy of both treatments in reversing RV dysfunction in acute PE, with EKOS potentially offering a slight improvement over IV alteplase.

A recent study emphasized on the role of hemodynamic stability in improving both pulmonary and cardiac function in high-risk massive PE patients (Baran et al., 2024).

At the end of the follow-up, the outcomes for the studied groups showed that fewer patients who received EKOS experienced major bleeding events after 72 h compared to the control group (p = 0.03). However, there was no significant difference between the two groups in terms of major bleeding events within 6 months, all-cause mortality, or all-cause readmission within 30 days.

While EKOS showed fewer major bleeding events after 72 h, this finding did not translate into a survival benefit that influenced all-cause mortality. As both EKOS and IV alteplase achieved comparable improvements in RV function in PE, and without a significant difference in major bleeding events within 6 months, long-term outcomes, such as 6-month all-cause mortality, are expected to remain similar.

A recent retrospective cohort study evaluated the clinical outcomes of ultrasound-assisted CDT in intermediate- to high-risk acute and subacute PE. After the 6-month follow-up, all PE patients remained hemodynamically stable, with no signs of recurrent VTE or deterioration in cardiovascular function. In addition, they reported statistically significant improvements in ventricular function, making this treatment approach a safe and clinically effective option, thus paving the way for its preference in clinical practice (Karaçuha et al., 2025).

Kucher et al. found that a standardized ultrasound-assisted thrombolysis regimen was effective in reversing RV dilatation within 24 h without increasing the risk of bleeding complications, with a significant decrease in the mean RV/LV ratio from 1.28 ± 0.19 to 0.99 ± 0.17 at 24 h. Additionally, only three minor bleeding episodes occurred, and there were no reported cases of recurrent VTE (Kucher et al., 2014).

Moreover, Piazza et al. demonstrated that ultrasound-facilitated, catheter-directed, low-dose fibrinolysis effectively reduced RV dilation, pulmonary hypertension, and anatomic thrombus burden in patients with acute massive and sub-massive PE, with a significant decrease in the mean RV/LV ratio from 1.55 to 1.13 at 48 h post-procedure. Furthermore, this treatment approach was associated with a low risk of intracranial hemorrhage (Piazza et al., 2015).

Tapson et al. showed that treatment with ultrasound-facilitated CDT using a shorter delivery duration and lower-dose tPA was associated with improved RV function and reduced clot burden compared with baseline (Tapson et al., 2018).

When choosing a treatment approach for patients with PE, it is crucial to consider additional factors beyond the patient’s condition. Specifically, for patients who have absolute contraindications to fibrinolytic therapy, EKOS is not a viable option (Tu et al., 2019).

One advantage of the EKOS system is its relatively short average procedure time, 46 ± 6.2 min in the present study. In contrast, the advanced thrombectomy devices, such as the FlowTriever system, had a mean procedure time of 93.8 min. The shorter procedure time associated with EKOS reduces the patient’s risk of complications and infection. Additionally, EKOS is a more cost-effective option compared to the FlowTriever system, which is significantly more expensive (Mangi et al., 2017; Avgerinos et al., 2021).

Given that ultrasound-assisted thrombolysis is 10 times more costly than standard CDT, the benefit of ultrasound-assisted thrombolysis may not be worth the cost.

Sun et al. did not detect any differences in all-cause mortality, total bleeding, or major bleeding events between non-low-risk patients with PE treated with ultrasound-assisted thrombolysis and those treated with standard CDT (Sun et al., 2022).

In the SUNSET trial, they compared traditional CDT to ultrasound-assisted thrombolysis using similar mean lytic doses and durations of lysis. Patients receiving USAT exhibited comparable reductions in pulmonary arterial thrombus, though ultrasound-assisted thrombolysis achieved a significantly greater reduction in the RV/LV ratio than USAT, suggesting a probable higher efficacy in relieving RV overload. In addition, both groups were comparable in terms of safety profiles, as assessed by major and minor bleeding, ICU stay, and in-hospital mortality rates. The actual significance of EKOS may lie in reducing the dose and duration of thrombolytic therapy to improve patient outcomes (Avgerinos et al., 2021).

The univariate analysis revealed only one significant predictor (early hemoglobin decline ≥ 2 g/dL within 72 h), while all other variables were non-significant. Given the low event rate and the risk of overfitting, multivariate adjustment would yield unreliable and uninterpretable estimates. Therefore, results were presented as univariate exploratory associations.

These findings imply that bleeding-related complications and early hemoglobin decline are the main determinants of mortality following thrombolytic therapy for PE. This result is consistent with large registry data (e.g., PEITHO and ICOPER), where major bleeding events independently predicted in-hospital mortality in PE patients treated with fibrinolytics. Studies reported by Kucher et al. and Tapson et al. also showed that while CDT improves RV function, it does not necessarily reduce long-term mortality, supporting the non-significant results of EKOS vs. alteplase in our study (Kucher et al., 2014; Tapson et al., 2018).

Both baseline and post-treatment echocardiographic parameters, including TAPSE, RV velocity, and RV/LV ratio, showed no significant association with mortality, a finding that likely reflects several interacting factors. First, fewer deaths were reported, limiting statistical power to detect the prognostic effects. Second, all patients had intermediate- to high-risk PE and underwent successful reperfusion (EKOS or systemic alteplase), which rapidly improved RV function and minimized residual hemodynamic differences between survivors and non-survivors. Third, most deaths were related to bleeding rather than right-heart failure, reducing the impact of RV indices on the outcome.

These results are consistent with prior multicenter data showing that ultrasound-assisted CDT improves RV function without conferring a clear survival benefit. Kucher et al. demonstrated the reversal of RV dilatation in the ULTIMA trial without mortality reduction (Kucher et al., 2014), while Tapson et al. (OPTALYSE PE) reported similar findings of rapid RV recovery with low major bleeding rates but unchanged long-term survival (Tapson et al., 2018). Avgerinos et al., likewise, found no mortality difference between ultrasound-assisted CDT and standard CDT (Avgerinos et al., 2021), and Draxler et al. confirmed that RV functional improvement does not necessarily translate into reduced number of deaths (Draxler et al., 2025). Collectively, these studies and our findings indicate that TAPSE and the RV/LV ratio serve primarily as short-term markers of therapeutic response rather than independent mortality predictors once effective reperfusion has corrected RV strain.

In our study, no baseline clinical, hemodynamic, or echocardiographic variables independently predicted major bleeding events, and the treatment modality (EKOS vs. systemic alteplase) comparison yielded non-statistically significant results, although the absolute bleeding incidence was lower among EKOS-treated patients (3.7% vs. 16%). The borderline association detected for baseline TAPSE was accompanied by an extremely wide CI, reflecting sparse-event bias, and should be regarded as a statistical artifact rather than a physiologic relationship. These findings indicate that the risk of hemorrhage during thrombolytic therapy for PE is largely treatment-related rather than patient-specific, which is consistent with prior evidence that systemic fibrinolytic exposure, rather than the baseline cardiopulmonary status, determines bleeding propensity.

Multiple randomized and prospective trials have demonstrated that ultrasound-assisted CDT using markedly lower recombinant tissue-plasminogen activator (rt-PA) doses achieves rapid reversal of RV dysfunction with an excellent safety profile. The pivotal ULTIMA trial showed that ultrasound-assisted CDT plus heparin therapy rapidly normalized the RV/LV ratio within 24 h without any major bleeding events (Kucher et al., 2014).

The larger, multicenter SEATTLE II study confirmed significant reductions in RV dilatation and pulmonary-artery pressure using 24 mg total tPA, reporting a 10% major-bleeding rate but no intracranial hemorrhage (Piazza et al., 2015). Subsequently, the OPTALYSE PE trial demonstrated that even ultra-low-dose, short-duration regimens (8 mg–24 mg infused over 2 h–6 h) provided equivalent RV recovery with only ∼4% major bleeding events and one intracranial bleeding event (Tapson et al., 2018). Collectively, these studies established that ultrasound-assisted CDT can significantly lower hemorrhagic risk compared with systemic thrombolysis, primarily by limiting total fibrinolytic exposure.

Our observation that bleeding rates did not differ statistically between EKOS and systemic alteplase, despite a numerical advantage for EKOS (16 % vs. 3.7 % for EKOS vs. systemic alteplase), mirrors the results from the SUNSET sPE randomized trial, which compared ultrasound-assisted and standard CDT and found similar efficacy and reported no intracranial or fatal hemorrhages. The current findings, therefore, reinforce the concept that bleeding risk in reperfusion therapy for PE is determined predominantly by the thrombolytic strategy and dose, not by patient comorbidity or RV function, and that ultrasound-assisted CDT offers a favorable safety–efficacy balance compared with systemic alteplase in appropriately selected intermediate- to high-risk PE patients.

## 5 Limitations

This study has several significant limitations that should be acknowledged: its retrospective observational design resulted in a lack of randomization, which introduces potential selection bias. Considering the safety profile, some data were missing from the patient’s profile, including information on minor bleeding events.

As the lack of randomization and blinding may raise concerns about the confounding factors, we performed univariate and multivariate analyses to control for potential confounders.

Moreover, the study was conducted in a single center, which limits the generalizability of the findings as clinical decisions may have been influenced by operator preference and limited resource availability.

The limited sample size reduces the precision of effect estimates, which may hinder the detection of significant differences in hard clinical outcomes, such as mortality. It was not feasible to assess the economic impact of the EKOS system despite its importance. Therefore, the current findings should be interpreted as exploratory and hypothesis-generating. However, the study remains valuable in adding real-world evidence from a regional setting where data on CDT are scarce. Importantly, it provides a foundation for designing future multicenter, prospective, adequately powered trials to confirm these findings and further refine the patient selection criteria for EKOS therapy in cases of acute PE.

## 6 Conclusion

This study compares the effectiveness and safety of the EKOS intervention with that of IV alteplase in reducing right heart strain and minimizing complications in patients with PE who are at an intermediate to high risk of failure with anticoagulation therapy. The findings contribute to the growing body of evidence supporting the use of EKOS. The present study showed that the RV/LV diameter ratio was significantly decreased, while TAPSE was significantly enhanced in the EKOS and control groups. The EKOS group had fewer patients suffering from major bleeding events after 72 h than the control group. Moreover, after 6 months, the EKOS group experienced a lower rate of all-cause mortality compared to the control group, with no significant difference between the groups.

As the management of PE continues to evolve, EKOS provides healthcare providers with an additional tool to improve patient outcomes. However, the decision to use EKOS should be made on a case-by-case basis, taking into account the institutional resources, provider expertise, and the patient’s clinical situation.

## Data Availability

The raw data supporting the conclusions of this article will be made available by the authors upon reasonable request, without undue reservation.
