# Supplementary material for: Real-world clinical outcomes of EKOS catheter-directed thrombolysis versus systemic alteplase in acute pulmonary embolism: a retrospective cohort study
Source: Front Pharmacol. 2026 Jan 6;16:1709708. doi: 10.3389/fphar.2025.1709708 (PMC12816243; doi:10.3389/fphar.2025.1709708)
Supplement: Supplementary file 1 [file Table1.pdf]

Supplementary Table 1: Improvement of NEWS Score Parameters Before and After Therapy in Pulmonary Embolism Patients

Variable	EKOS (Before)	EKOS (After 48 hr)	Alteplase (Before)	Alteplase (After 48 hr)	p-value	Interpretation
Respiratory rate (cycles/min)	24	10	24	10	0.90	Marked improvement in both groups
SpO_2 (%)	85 %	90 %	85 %	90 %	0.99	Improved oxygenation
O_2 supplementation required	Yes	Yes	Yes	Yes	1.00	No significant change
Temperature (°C)	36.3	36.0	36.2	36.1	0.85	Stable
Systolic BP (mm Hg)	90	95	90	93	0.72	Slight improvement
Pulse (beats/min)	110	95	110	93	0.68	Improved heart rate
Level of consciousness	Conscious	Conscious	Conscious	Conscious	1.00	Stable
**Total NEWS score**	**12**	**9**	**12**	**9**	**0.95***	**Numerical improvement, not statistically significant***
